# Technical considerations of functional MRI in deep brain stimulation: recent advances and future perspectives

**DOI:** 10.1016/j.neuroimage.2026.121889

**Published:** 2026-03-25

**Authors:** Andrej Lasica, Silvia Mangia, Shalom Michaeli, Manuela Vaneckova, Robert Jech, Pavel Filip

**Affiliations:** a Department of Neurology, Charles University, First Faculty of Medicine and General University Hospital, Prague, Czech Republic; b Center for Magnetic Resonance Research (CMRR), University of Minnesota, Minneapolis, MN, USA; c Department of Cybernetics, Czech Technical University in Prague, Prague, Czech Republic; d Department of Radiology, First Faculty of Medicine, Charles University and General University Hospital, Prague, Czech Republic

**Keywords:** Functional magnetic resonance imaging, Deep brain stimulation, Imaging artefacts, Imaging safety

## Abstract

Magnetic resonance imaging (MRI) is a valuable clinical and research tool for patients managed using deep brain stimulation (DBS). Unfortunately, MRI under these conditions is associated with substantial risks, necessitating stringent regulations that limit its clinical and research utility. In addition, magnetic susceptibility differences between DBS hardware and surrounding tissues significantly compromise spatial encoding mechanisms of conventional MRI sequences, resulting in image signal loss and geometric distortions. The impact on gradient-recalled echo echo-planar imaging sequences commonly utilised for functional MRI in DBS settings is particularly severe. This review presents a range of mitigation strategies aimed at both safety and enhanced image quality, spanning innovations in DBS hardware design to advanced MRI sequences capable of addressing issues inherent to the presence of DBS hardware, such as electrode heating and susceptibility artefacts. Additionally, we highlight approaches incorporating the discussed novel postprocessing techniques and functional MRI acquisition protocols, along with their limitations and associated challenges to enable their wider dissemination, with the overarching objective of improving the quality of life of DBS patients.

## 1. Introduction

Deep brain stimulation (DBS) is an increasingly popular intervention employing electric current to modulate the activity in specific brain regions via permanently implanted electrodes. DBS has been shown to elicit meaningful therapeutic effects in motor disorders and epilepsy, with promises also for patients with cognitive impairments and psychiatric conditions (Lozano et al., 2019). Nevertheless, DBS as a method is not devoid of major perils, be it suboptimal efficacy, adverse effects related to inaccuracies in electrode placement or inadequately refined DBS parameters. As a result, there is a pressing need for non-invasive measurement methods that can monitor the brain at both structural and functional level, before and after implantation, to evaluate the effects of DBS on neural circuitry.

While the advent of new hardware advances allowing for continuous monitoring of local field potentials directly within the electrode area (Medtronic, 2025) has partly answered this call, a more flexible tool providing a larger overview at the level of relevant networks or even the whole brain is necessary. Magnetic resonance imaging (MRI) is uniquely positioned to meet this requirement owing to the absence of ionising radiation, acceptable spatial resolution, and flexibility for non-invasive generation of multiple image contrasts. Particularly functional MRI (fMRI) has been proposed as a viable biomarker for the evaluation of engagement of relevant neural circuits and prediction of possible treatment efficacy, in part due to the observed correlation between fMRI signals and neuronal local field potentials (Loh et al., 2022, Shi et al., 2019). On the other hand, MRI also presents several notable drawbacks, including its high operational costs, relatively long acquisition times, and pronounced acoustical noise arising from gradient switching. In addition, power deposition induced by the radiofrequency irradiation during scanning constitutes an additional challenge of particular importance in DBS settings. The electrode can effectively act as a local antenna, thereby increasing the risk of tissue damage around the implant (Boutet et al., 2020). Furthermore, obtaining information from the vicinity of the electrode is particularly challenging, since the electrode introduces susceptibility artefacts leading to signal loss and/or significant image distortions whose magnitude depends not only on the electrode material, but also acquisition parameters.

The aim of this review is three-fold. First, it seeks to familiarise the reader with the current state of DBS fMRI research. Secondly, it examines the obstacles associated with MRI in DBS applications, with particular focus on safety and image artefacts in fMRI, while also discussing the measures taken by manufacturers to render electrodes compatible with MRI environment. Thirdly, it elucidates the most promising MRI sequences currently available for imaging the brain with implanted DBS electrodes, describing their principles, advantages, limitations, common features, differences and suitability for fMRI applications. Although concise, these descriptions are offered as a preliminary foundation to build upon in further, more in-depth exploration, as outlined in its last section of the review containing a forward-looking perspective on DBS fMRI research, along with concluding observations.

## 2. Functional MRI in DBS: where are we now?

It has been over two decades since the initial pilot studies employing fMRI in DBS (Rezai et al., 1999, Jech et al., 2001, Stefurak et al., 2003). In those early endeavours, neurostimulators were incompatible with MRI scanners, prompting investigators to perform fMRI perioperatively, using externalised stimulators controlled from a safe distance. With the advent of MRI-conditional neurostimulators as well as the demonstration of safety of fMRI with DBS under controlled conditions (Boutet et al., 2020), an increasing number of researchers began to focus on DBS fMRI studies. This, in turn, led to broader interest across multiple centres and recruitment of more patients. Nonetheless, the number of study participants remains relatively modest – usually in the range of 10-20, significantly affecting the generalisability and replicability of results, due to the small population of DBS patients in each DBS centre and device compatibility (Jech and Mueller, 2022). Designing studies around these limitations is an important aspect of DBS research.

Additionally, the majority of clinical DBS fMRI research has been conducted at 1.5T MRI scanners, with only a limited number of studies performed at 3T (Jech and Mueller, 2022, Boutet et al., 2021, Bohara et al., 2023, Iskin et al., 2025, Qiu et al., 2024). Research methodologies vary considerably, encompassing resting-state fMRI with cyclical on/off block design (Boutet et al., 2021, Bohara et al., 2023, Iskin et al., 2025, Qiu et al., 2024, DiMarzio et al., 2020, DiMarzio et al., 2021, Hancu et al., 2019, Shen et al., 2020), switching DBS on and off between separate runs (Horn et al., 2019, Hanssen et al., 2019, Mueller et al., 2018, Kahan et al., 2019, Kahan et al., 2014, Mueller et al., 2013, Kahan et al., 2012), and task fMRI coupled to voluntary actions or stimuli (e.g. finger tapping) in block-design paradigms (Mueller et al., 2020, DiMarzio et al., 2019, Jech et al., 2012). Moreover, the generalisability of findings is further hindered by the spectrum of neuropsychiatric conditions and hence anatomical targets, where DBS may be employed. Most investigations continue to focus on the effect of DBS in patients with Parkinson’s disease and other disorders like essential tremor, dystonia and epilepsy remain under-reported due to the relative scarcity of relevant patient populations (Loh et al., 2022). Nonetheless, DBS effects seem to share a common, overarching pattern: normalising the activity and connectivity towards the state detected in healthy population (Horn et al., 2019, Saenger et al., 2017, Filip et al., 2022). This concept has been repeatedly demonstrated in several DBS anatomical targets (Filip et al., 2022, Filip et al., 2025) and neural networks – not only of sensorimotor function, but also those associated with cognition and mood (Filip et al., 2024). However, published studies are far from devoid of conflicting reports on the direction of DBS-elicited connectivity changes (Hancu et al., 2019, Shen et al., 2020, Knight et al., 2015), thus complicating their interpretation (Rezai et al., 1999, Gibson et al., 2016, Awad et al., 2020).

Due to the complexity of the DBS stimulation settings, testing across segmented lead configurations in every patient is not feasible due to time constraints and is therefore typically reserved for cases in which standard settings fail to provide sufficient clinical benefit. The advent of adaptive stimulators, which are able to vary amplitude in real time based on electrophysiological markers, has created a parameter space of thousands of possible settings (Neumann et al., 2023), further reinforcing the need for at least partial automatization of DBS titration. Machine-learning classifiers such as autoencoder-based feature extraction or probabilistic models (Roediger et al., 2022) coupled with characteristic fMRI network signatures of clinically optimal stimulation settings have been shown to predict best contact-amplitude combination for individual patients (Boutet et al., 2021, Roediger et al., 2022). This “first-pass” programming approach could then be utilised as the foundation for further conventional titration by DBS experts or eventually as a fully automated pipeline for clinician-independent DBS optimisation (Torres et al., 2024). Although further refinement is required, fMRI was already successfully used in a preliminary subset of patients to revert the cognitive deterioration which had developed as a side-effect of DBS (Reich et al., 2022), and as a treatment modality using neurofeedback paradigm (Baqapuri et al., 2025). The selection of the right region of interest is critical for this modality, and an improved understanding of local DBS effects could further enhance its efficacy (Baqapuri et al., 2025).

As noted previously, DBS influences both local and distributed brain networks (Ashkan et al., 2017). Nevertheless, recent invasive neurophysiological studies point to the importance of local DBS effect compared to distant effects through, for example, hyper-direct pathway (Neumann, 2022). After an initial period during which DBS effect is mediated by neurotransmitter-dependent activation of afferent axons, stimulation induces a long-term synaptic depression akin to reversible lesion of the stimulated nucleus (Milosevic et al., 2018, Milosevic et al., 2018, Milosevic et al., 2019, Milosevic et al., 2019). Importantly, areas in the immediate vicinity of the electrode may also constitute components of broader functional brain networks, but their contribution is difficult to assess using conventional fMRI, leaving previously published network-level alterations potentially biased towards distal effects (Manes et al., 2014, Manes et al., 2018). In turn, DBS might affect distant networks by changing the activity of one out of many nodes in the network, thereby altering the activity of other nodes in the same network (Neumann, 2022). These remote effects can still provide valuable information about network engagement, but do not allow for direct inference from local peri-electrode region central to clinical DBS effects (Neumann, 2022, Alhourani et al., 2015). Moreover, Electrophysiological studies indicate that neuronal firing patterns in the subthalamic nucleus and internal pallidum show substantial alterations in Parkinson’s disease (Galati et al., 2006, Lee et al., 2021). Distal network effects enhanced with reversal of these local changes could therefore serve as a potential biomarker of DBS efficacy, together with increasing the accuracy of imaging feature-based machine learning algorithms (Boutet et al., 2021, Qiu et al., 2024, Germann et al., 2024). However, despite their potential importance, local effects of DBS remain understudied due to imaging-related limitations and represent an important target for future research.

## 3. Challenges for MRI and fMRI in DBS applications

**Fig. 1.**
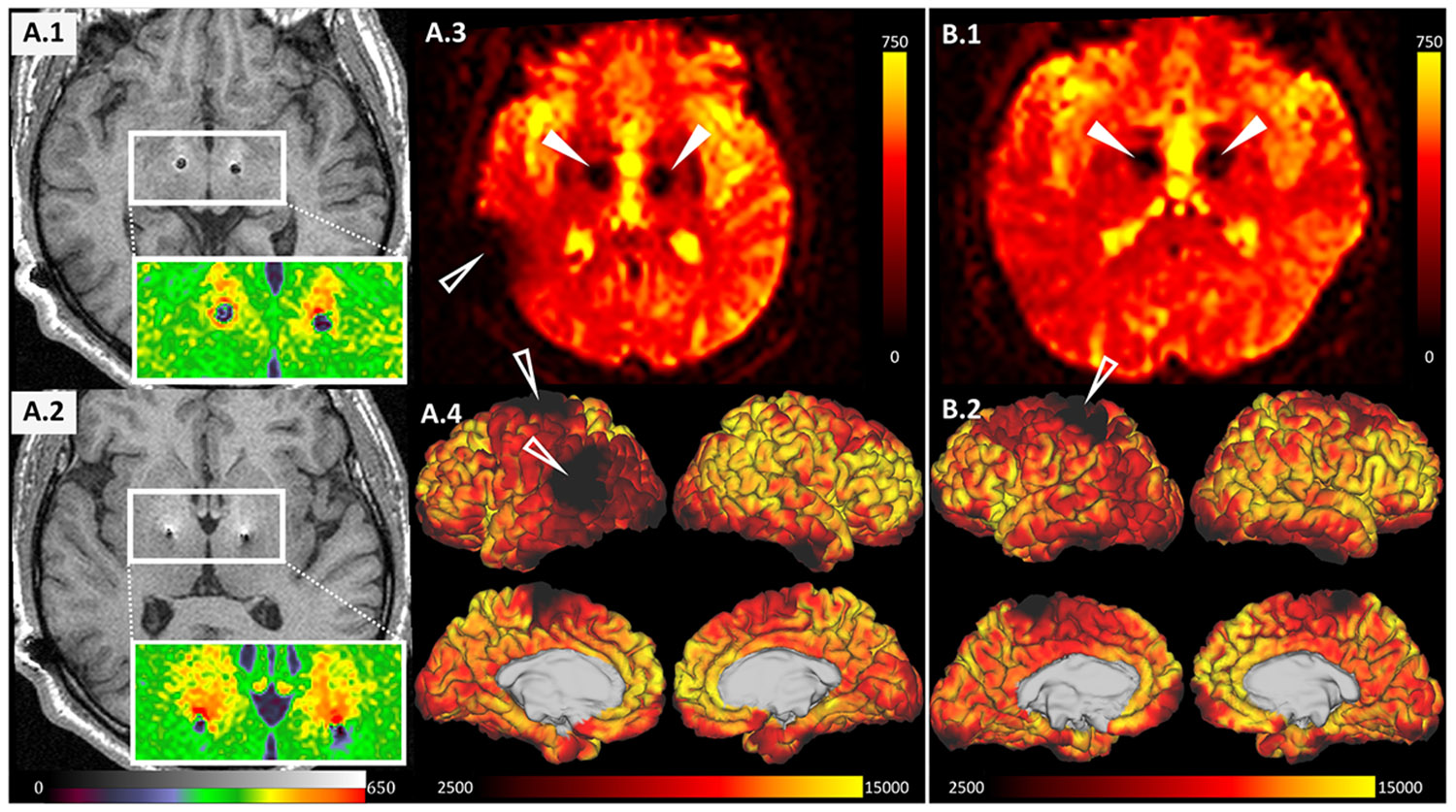
Extent and location of DBS-hardware-related susceptibility artefacts in MRI scans. 1.5 MAGNETOM Avanto scanner, 2 patients with Parkinson’s disease (A and B) with electrodes implanted in the subthalamic nucleus. **A.1 and A.2** – T_1_-weighted magnetisation-prepared rapid gradient echoes (MPRAGE) sequence, axial plane (A.1) at the level of electrode contacts showing ring-like hyperintensity and about 6 mm wide signal drop-out, and (A.2) at a higher level with encased electrode segment, depicting smaller area of signal drop-out, but extensive surrounding hyperintensity (see zoomed-in views of white frames in colour-scale for better visualisation). **A.3 and A.4** – Gradient-recalled echo echo-planar imaging (GRE-EPI) sequence, A.3 at the level corresponding to A.1, depicting substantially larger signal drop-out in the vicinity of the electrode (10-15 mm), but also cortical signal drop-out in the left temporo-occipito-parietal junction related to subcutaneous DBS hardware (extension wire and adapters), better visible in A.4 – fMRI signal fitted to cortical surface reconstruction using Human Connectome Project pipeline (Glasser et al., 2013). White arrowheads mark subcortical signal dropouts around the implanted electrodes, empty arrowheads point to cortical signal dropouts mostly around extension wire adapters. **B.1 and B.2 –** GRE-EPI acquisition of a different patient with DBS electrodes, with substantially lower signal dropouts in the cortical region (B.2) compared to patient A, despite having the same hardware configuration, but unintentionally slightly different extension wire adapter position. Laterality conventions where left side of the patient corresponds to the left side of the scan was utilised.

MRI relies on the use of a strong magnetic field to generate images of objects placed within the scanner bore. First and foremost, safety issues as magnetic field interactions with DBS hardware leading to component migration and induced electrical currents with subsequent tissue heating and potential disruption of device operation must be considered (Boutet et al., 2020). Secondly, the introduction of metallic objects into the imaging volume inherently results in susceptibility artefacts at high magnetic fields, whose properties depend on both the shape and magnetic susceptibility of the implanted object. Collectively, artefacts around DBS hardware render large portions of the brain invisible to fMRI. These artefacts are not confined to the stimulation region, as for instance subthalamic nucleus or internal pallidum, but extend into white matter tracts, deep grey matter structures and cortical regions traversed by the electrode (Fig. 1). Moreover, the residual length of the wire coiled beneath the scalp can obscure a substantial part of the cortex.

### 3.1. Safety issues

Hardware dysfunction and possible MRI field-induced device migration could have been considered a major issue in older neurostimulator models containing ferromagnetic material such as ferrite core antennae or even magnetic reed switches to turn the device on and off (Gleason et al., 1992).The commonly used newer, MRI-conditional components are mostly nonmagnetic or diamagnetic (Abbott Ap 2025, Boston Scientific M 2026, Medtronic, 2022), greatly reducing mechanical forces or MRI field effects on the hardware itself.

However, even in MRI-conditional hardware, there is a high risk of acquisition-induced currents in DBS equipment, specifically via two key mechanisms: impulses induced by time-varying magnetic fields generated by radiofrequency (RF) pulses and gradient switching, and currents induced by antenna effect (Georgi et al., 2004). The carrier frequency of RF currents in megahertz range is unlikely to elicit neuronal activity, but gradient switching producing up to 1.5 V may induce lower frequencies overlapping with the neuronal firing range (Kahan et al., 2015). Nevertheless, these low voltages are unlikely to cause major patient discomfort given the electrode position in clinically relevant therapeutic targets (Ham et al., 1997). In addition, the induced voltage magnitude is comparable to stimulation levels employed in the clinical settings, even in case of their occasional superimposition upon neurostimulator impulses. However, even this modest induced voltage and the corresponding elicited neuronal activity may act as a non-negligible confounder in studies employing fMRI to contrast on versus off DBS states (Loh et al., 2022). Tissue heating due to gradient-induced eddy currents is generally considered a minor contribution relative to RF-induced heating given the shape and effective area of induced current loops in standard DBS leads (Winter et al., 2021, Arduino et al., 2022). Possible additional mitigation strategies may include reducing the gradient switching speed – an established feature of sequences described below (Winter et al., 2021).

On the other hand, while RF excitation pulses operating in the megahertz domain should not elicit neuronal firing, induced voltage reaches far higher levels of up to 7.0 V (Georgi et al., 2004). This energy is ultimately dissipated as heat at the location with greatest electrical current flux density – the electrode tip. Rat studies show that not only does this phenomenon carry the risk of brain tissue damage, but even temperature increases within 2°C (Finelli et al., 2002) deemed acceptable and within established safety margins may alter the blood oxygen level dependent (BOLD) signal and ultimately fMRI data quality (Harris et al., 2018).

**Fig. 2.**
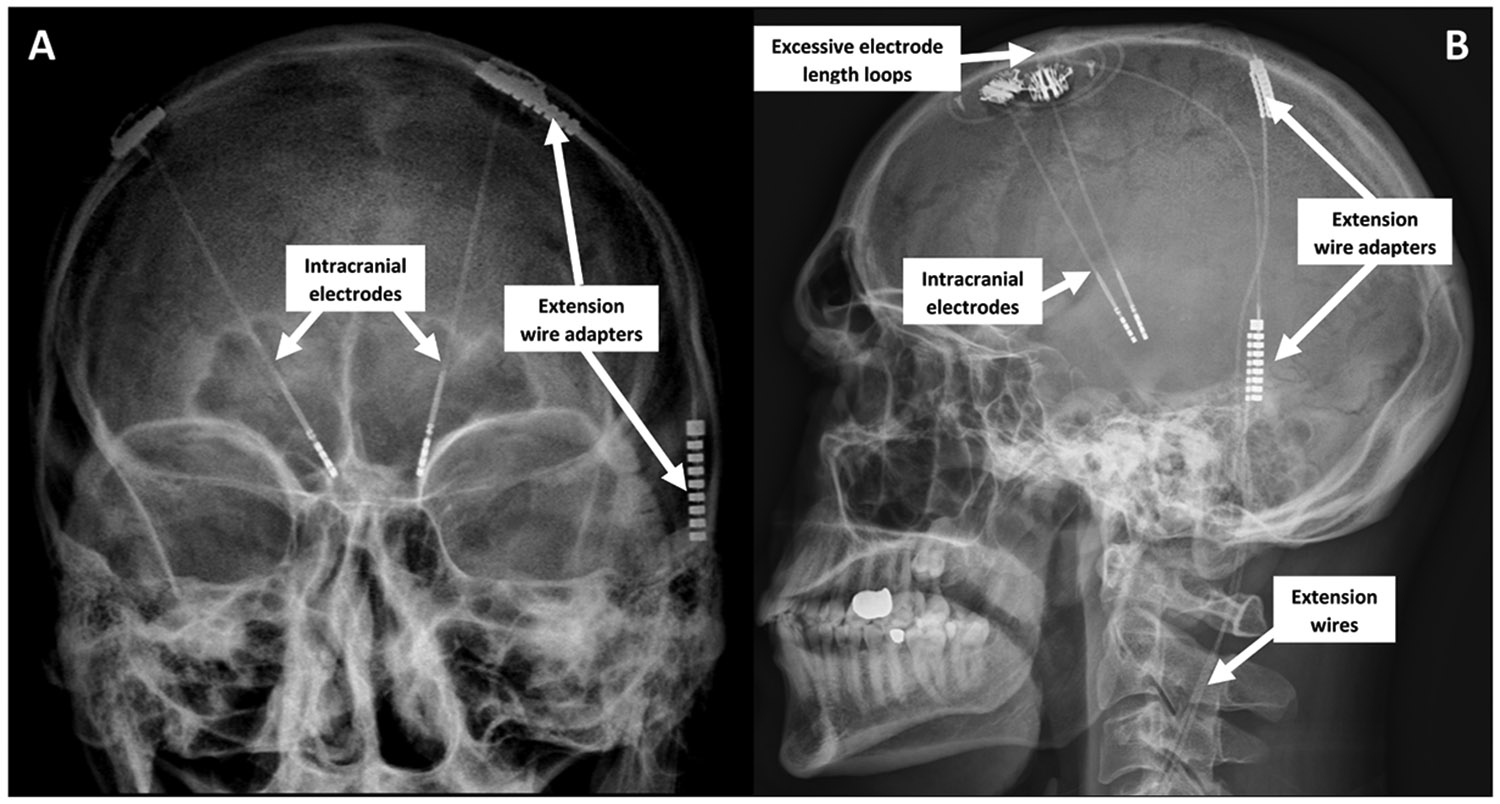
X-ray scan of a patient with implanted DBS system showing the full intracranial (leads) and scalp region hardware configuration (loops of excessive electrode length, adapters and extension wire), as possible sources of susceptibility artefacts. A – anteroposterior and B – lateral view.

The principal external determinant of the RF-related heating of DBS hardware is the magnitude of applied RF energy and associated heating-related thresholds – the specific absorption rate (SAR) and the rootmean-square of the MRI effective component of the RF magnetic (B_1_) field (B_1_+RMS) (Oluigbo and Rezai, 2013). Intrinsic factors include the type of neurostimulator and implanted hardware geometry, which will be discussed below (see Fig. 2 for X-ray scans depicting a common configuration of implanted hardware components) (Gupte et al., 2011). Altogether, despite the generally strict precautions employed in patients with neurostimulators, there have been five documented cases of injuries associated with RF currents, three of which occurred during MRI examinations (Spiegel et al., 2003, Henderson et al., 2005, Zrinzo et al., 2011).

Imaging patients with bipolar settings is generally safer compared to monopolar settings due to the smaller size of the current loop in bipolar configurations (Wu et al., 2026). Hence, most manufacturers do not approve monopolar stimulation during MRI, and on-label research protocols require patients to be reprogrammed to bipolar settings (Jech and Mueller, 2022, Hancu et al., 2019, Abbott Ap 2025, Boston Scientific M 2026, Medtronic, 2022). However, while bipolar stimulation provides a safer imaging environment, it produces a modulation effect that differs demonstrably from the ideal monopolar configuration (Hancu et al., 2019, Wu et al., 2026).

### 3.2. Susceptibility artefacts around metallic implants

Magnetic susceptibility is a measure of the extent of magnetisation of any substance relative to an applied external magnetic field. While a crude oversimplification, MRI as utilised in clinical settings may be considered to primarily image protons in water, with the susceptibility of −9 × 10^−6^ at 37°C scaling linearly with temperature (Gutiérrez-Mejía and Ruiz-Suarez, 2012). Consequently, the presence of any material with a magnetic susceptibility different from that of water has the potential to introduce so-called susceptibility artefacts, as evident for instance in air-tissue interfaces exhibiting non-negligible interference with fMRI signals even in subjects devoid of any brain implants. Magnetic field distortions induced by susceptibility effects lead to variations in precessional Larmor frequency, causing signal attenuation due to T_2_*-dephasing and incorrect mapping of the MRI signal in space. Consequently, the resulting MRI artefact emerges as the combination of both these physical mechanisms: geometric distortion accompanied by localised areas of signal loss, as well as regions with apparent increased intensities due to signal being erroneously attributed to and cumulated in the wrong areas.

**Fig. 3.**
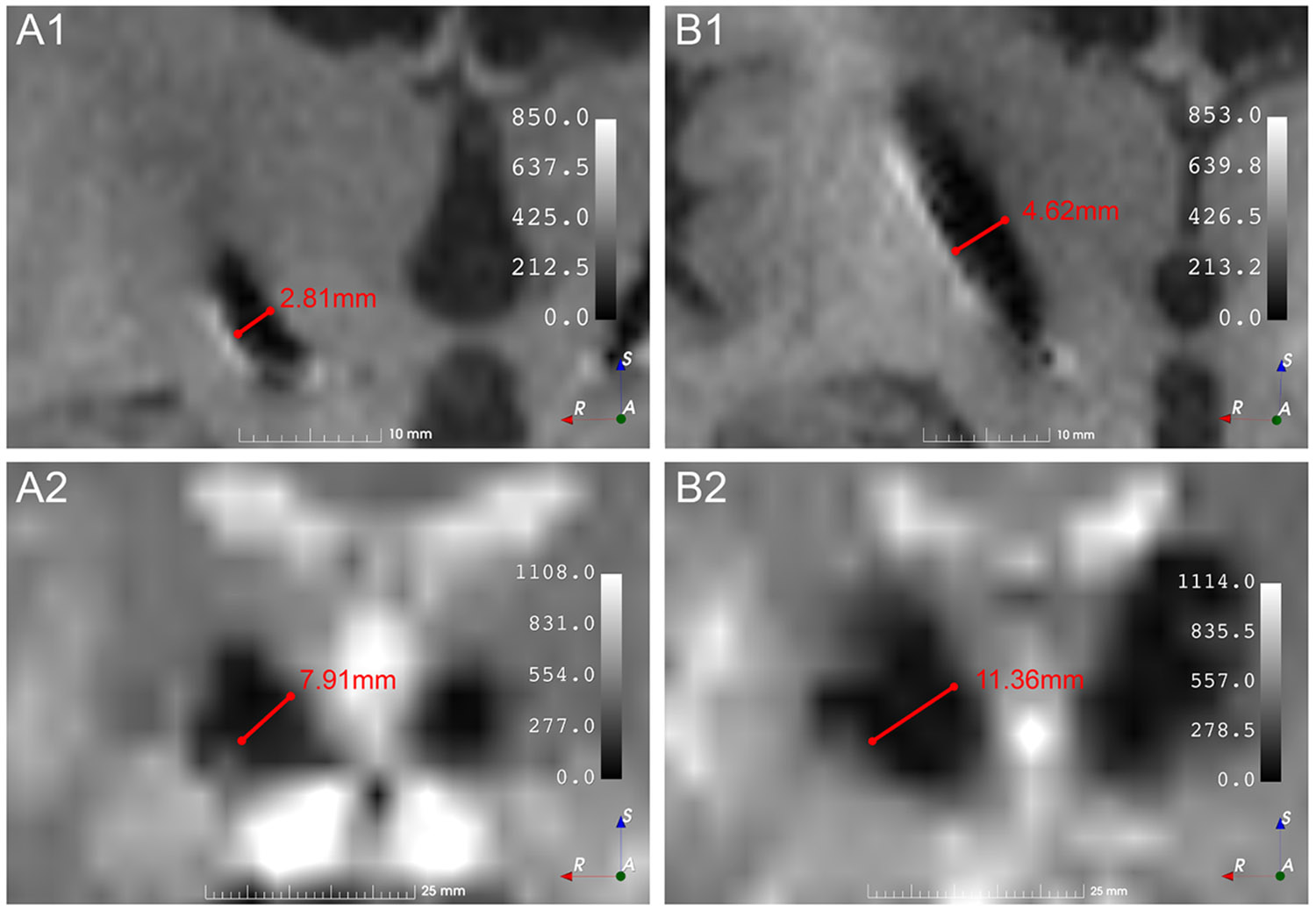
Comparison of artefact size for DBS electrode models 3389 manufactured by Medtronic (A) and 6172 manufactured by Abbott (B) on T1 MPRAGE (A1, and B1) and gradient-echo EPI fMRI image (A2, and B2). All images are shown in a coronal view, zoomed in on the right basal ganglia. The MRI scanner and scanning parameters used for these acquisitions were identical, with the two scans acquired approximately two months apart. For detailed imaging parameters, please refer to our previous work (Filip et al., 2024, Lasica et al., 2025).

Since ferromagnetic materials have the highest magnetic susceptibilities, substantial field distortions associated with MRI artefacts commonly occur around metallic objects and implants such as DBS electrodes. Their shape and intensity strongly depend not only on scanner parameters as MRI field strength, echo time (TE) and bandwidth, but also on the electrode material, its shape and orientation relative to the applied magnetic field (see Fig. 1 depicting the area of signal loss and apparent intensity increase in the vicinity of DBS electrode) (Bakker et al., 1994, Li et al., 1995). Consequently, rapid MRI sequences such as gradient-recalled echo echo-planar imaging (GRE-EPI), routinely employed in fMRI research, are particularly vulnerable to these off-resonance effects, as they do not incorporate refocusing pulses (Poustchi-Amin et al., 2001, Farahani et al., 1990). As a result, fMRI signal attenuation and spatial shifts reach substantial extents that can impact reliability of inference in peri-electrode regions, documented by lower retest reliability measured by intraclass correlation coefficient in artefact affected parcels compared to unaffected parcels (see Figs. 1, and 3 for susceptibility-related artefact in the vicinity of the DBS lead, as well as in the lower parietal cortex beneath the subcutaneous DBS hardware) (Hargreaves et al., 2011, Deutsch et al., 2026). Unfortunately, conventional measures such as the incorporation of field maps or increasing the performance of phase-encoding gradients generally prove insufficient for satisfactory correction (Smith, 2010, Reber et al., 1998, Wang et al., 2017).

## 4. Mitigation strategies

Guidelines intended to minimise patient risk in clinical practice have been the subject of several reviews (Boutet et al., 2020, Gupte et al., 2011, Martin, 2019). Nonetheless, while rigorous adherence to DBS MRI safety vendor recommendations generally ensures patient safety, it also restricts more sophisticated diagnostic assessments – not only the precise position of implanted electrodes, but the broader application of MRI as a comprehensive tool to elucidate structural and functional connectivity of individual contacts and thereby infer both the short- and long-term effects of DBS programming. For example, MRI tractography, among other advanced sequences, is technically feasible in DBS patients, yet the achievable image quality is severely limited due to concerns about tissue heating (Muller et al., 2020, Basich-Pease et al., 2023, van Hartevelt et al., 2015). Additionally, it restricts the use of MRI as a diagnostic tool for symptoms of other comorbidities later in life. Generally, susceptibility artefact mitigation strategies can be grouped into 3 main categories: (Lozano et al., 2019) the development of new materials or MRI sequences that reduce artefact size; (Medtronic, 2025) the use of post-processing algorithms for artefact correction; and (Loh et al., 2022) removing the voxels affected by artefact, which might leave large areas of brain unusable (Fig. 1), with different voxels being affected in each subject creating a problem of missing data (Boutet et al., 2019, Vaden et al., 2012, Mulugeta et al., 2017). Several approaches have been proposed in the literature to address the handling of the affected voxels, including coarse masks to exclude surface voxels and intensity-based thresholding with or without subsequent region averaging (Horn et al., 2019, Mueller et al., 2018, Zhang et al., 2021). The first and the second categories will be further expanded in this chapter.

### 4.1. Neurostimulator hardware material

All major manufacturers of DBS systems currently offer only MRI-conditional hardware (Abbott Ap 2025, Boston Scientific M 2026, Medtronic, 2022). The primary conditions relate to the strength of the main magnetic field, RF coil design, RF pulse strength, gradient slew rate and maximum gradient field strength. The limitation imposed on the main magnetic field strength is primarily attributable to the capacity of neurostimulators to withstand the strong magnetic fields. All neurostimulators currently on market are compatible with 1.5T MRI systems, with the sole exception of Medtronic Percept PC, which is also compatible with 3T MRI scanners. All systems are only compatible with circularly polarized head coil for brain imaging. The RF pulse strength constraint focuses on B_1+RMS_ of the field or, where this is not provided by the scanner, on the SAR value. Specific RF field strength requirements are summarized in Table 1. All three manufacturers limit the allowed slew rate at 200 T/m/s, with the maximum spatial field gradient strength ranging between 1900 and 4000 gauss/cm. Additionally, maximum scanning time for all major manufacturers is 30 minutes (not counting the idle time), with a 60 minutes rest time for Medtronic and Boston scientific, and 30 minutes rest time for Abbott (Abbott Ap 2025, Boston Scientific M 2026, Medtronic, 2022).

At present, all major DBS manufacturers employ platinum-iridium electrodes in their systems. The advantage of these materials lies in their biocompatibility and adequate charge-transfer mechanism (Cogan, 2008, Merrill, 2011). However, the magnetic susceptibility of platinum is approximately 270–280 × 10^−6^ (Schenck, 1996), and that of iridium is 37 × 10^−6^ (Lide, 2005). Since the susceptibility of water is −9 × 10^−6^ (Gutiérrez-Mejía and Ruiz-Suarez, 2012), this discrepancy causes the electrode artefact to cover a substantial area of brain tissue, pointing to a clear unmet need in the field. Moreover, despite using the same electrode material, different manufacturers produce electrodes with varying artefact sizes (see Fig. 3 for comparison of electrode artefact sizes between different manufacturers).

The most direct strategy to address the susceptibility artefacts would be to transition to a conductive material with the susceptibility close to that of water, namely copper, carbon nanotubes or graphene oxide. However, the strong cytotoxicity of copper (Stensaas and Stensaas, 1978) calls for relevant measures. Encapsulation of copper microwires in a protective graphene layer to prevent oxidation and thereby reduce cytotoxic properties has been shown as a viable solution, with negligible artefact compared to the platinum electrode (Zhao et al., 2016). An even better candidate could be found in carbon nanotubes: their conductive properties surpass those of platinum-iridium electrodes, they display exceptional biocompatibility, and their magnetic susceptibility closely approximates that of water (Lu et al., 2019, Zambrzycki et al., 2024, Jiang et al., 2011). Post-mortem histopathological examination revealed markedly reduced gliosis surrounding the electrode when compared to platinum-iridium, suggesting minimal inflammation and tissue damage beyond that caused by the implantation itself (Lu et al., 2019, Zambrzycki et al., 2024, Jiang et al., 2011). The artefact on T_2_-weighted images acquired using a 7T MRI scanner measured 268.4±29.9 μm for the carbon nanotube electrode, compared to 922.5±59.2 μm for the platinum-iridium electrode. Two drawbacks merit attention and possibly limit wide-spread use: the softness of the material precludes unaided penetration of brain tissue, and secondly, fibre orientation and manufacturing process exhibit substantial effects on the susceptibility of carbon nanotubes (Lu et al., 2019, Zambrzycki et al., 2024, Jiang et al., 2011). Graphene oxide is, like carbon nanotubes, relatively cheap, nontoxic, and can be readily integrated into complex microelectronic systems. The size and shape of graphene-based electrodes can also be tailored to each patient’s individual needs (Ria et al., 2025). Importantly, the size of artefacts on the morphological MRI scans is noticeably smaller compared with many conventional materials (Zhao et al., 2020). However, similar to carbon-nanotubes electrodes, the relative softness of graphene oxide electrode may pose challenges for tissue penetration. Of note there is also ongoing research in rats regarding other materials such as silver, and tungsten electrodes (Lai et al., 2015, Derksen et al., 2021).

The second strategy to achieve magnetic susceptibility comparable to that of water involves blending paramagnetic and diamagnetic materials to create a material with the desired susceptibility. This is exemplified by the gold-aluminium electrode. MRI experiments demonstrated that an electrode comprising of the combination of gold and aluminium microwires produced very limited perturbation of the B0 field, an artefact measuring 750 μm wide at ultrahigh field 16.4 T, and susceptibility differences of approximately 0.6 × 10^−6^ (Cruttenden et al., 2021). A notable benefit of these materials lies in their apparent non-reactivity in cerebral tissue (Merrill, 2011, Stensaas and Stensaas, 1978). Nevertheless, further research is needed to assess both the biocompatibility and the commercial viability of all the aforementioned material solutions. We refer interested readers to a recent, more detailed review on this topic (Kodama et al., 2017, Zhang et al., 2021, Luan et al., Xie et al., 2015).

### 4.2. RF-induced heating and MRI sequences

Heating-related thresholds (SAR and B_1+RMS_) are generally outlined as measures for establishing precautionary guidelines to mitigate the risk of tissue injury. While both are subject to several shortcomings, be it the differences in calculations among MRI system manufacturers and assumptions imposed on human body for SAR, or the lack of peer-reviewed evidence for B_1+RMS_ (Boutet et al., 2020), their applicability is substantially diminished in the presence of DBS implants. By their very definition, SAR and B_1+RMS_ indicate energy deposition across the whole body of the patient, assuming uniform heating within the field of view and hence fail to capture the localised high-RF electric fields at the electrode tips (Qian et al., 2013).

SAR depends on the electrical conductivity of the tissue, body size, RF frequency, flip angle and duty cycle. Therefore, the adherence to recommended guidelines (see Table 1) may necessitate modifying MRI protocols by reducing the number of slices, flip angles or echoes or by increasing repetition times. Not only do these alterations often result in diminished image quality and possibly longer acquisition times, but expert oversight is also necessary to ensure relevant goals are met and regions of interest remain within the imaging field of view with sufficient information levels retained. Pulse sequences that employ less frequent RF pulses with small tip angles pulses such as GRE-based sequences utilised in typical fMRI acquisitions are generally deemed safe for individuals with DBS implants (Jech et al., 2001). In contrast sequences that utilise more frequent refocusing RF pulses such as spin-echo (SE) or fast spin-echo (FSE)-based sequences tend to deliver greater RF power and are therefore more likely to induce heating of the tissue (Allison and Yanasak, 2015).

One primary approach to mitigate SAR near the electrode involves reducing the bandwidth of the RF pulse required for the field of view excitation. Varying RF pulse homogeneity and SAR according to k-space position has been proposed, thereby reducing total SAR by up to 50% (Homann et al., 2011). Moreover, parallel transmit strategy may be employed to generate implant-friendly modes and to reduce SAR in the vicinity of DBS implants, while also constraining peak global SAR and achieving the desired flip angle with viable homogeneity (Eryaman et al., 2015). Subsequent refinements of this approach incorporating brief preparatory scans to calculate implant-friendly excitation patterns have enabled a broad range of MRI sequences, including T_1_-weighed, T_2_-weighted (turbo spin echo and SPACE dark fluid), and ultra-short echo time (UTE), with markedly reduced temperature increases, mitigated RF artefacts and enhanced image quality around the DBS electrode (Eryaman et al., 2019). Additional strategies for reducing SAR via MRI protocol adjustments may include also lowering the flip angle, although this may compromise the information derived from the acquired images (Qian et al., 2013). Finally, a distinctly different, but highly impractical approach may lie in redesigning linearly polarised birdcage coils to enable coil rotation, thereby shifting its region of zero electric field into alignment with DBS electrodes (Golestanirad et al., 2017).

Electrode geometry plays an important role in RF-induced tissue heating. Substantial SAR differences between contralateral and ipsilateral configurations have been reported (ipsilateral being the side of the pulse generator implantation), with non-negligible effects of lead routing on this asymmetry (e.g. coiling the extension wire close to the burr hole to reduce the length of the initial straight segment) (McElcheran et al., 2019, Mattei et al., 2008, Golestanirad et al., 2019, Sadeghi--Tarakameh et al., 2022). In addition, novel lead technologies under development target heat dissipation heat along the lead length rather than concentrating it at the electrode tip (Serano et al., 2015, Bottomley et al., 2010). By contrast, the geometry of the electrode tip has received comparatively less attention showing reduction of the total energy dissipated in smaller contact sizes (Erhardt et al., 2019).

### 4.3. Susceptibility artefacts and MRI sequences

Several strategies may be employed to enhance image quality and reduce susceptibility artefacts in the vicinity of DBS electrodes. In comparatively simpler scenarios – such as imaging orthopaedic metal implants in tissues far less vulnerable to heating and RF-induced currents – optimisation may involve the use of smaller nominal voxel sizes (i.e. thinner slices and larger image matrices), higher receiver and RF pulse bandwidths, shorter echo times, and adjustments to the frequency and phase encoding directions (Feuerriegel and Sutter, 2024). FSE and short-tau inversion recovery (STIR) acquisition techniques, rather than single SE or GRE sequences, can further diminish susceptibility artefacts. Nevertheless, in patients with DBS implants, potential tissue heating and the risk of prohibitively exceeding SAR limits render such measures problematic, impractical, or even unfeasible in both clinical and research contexts. The necessary MRI sequence parameter adjustments would substantially reduce the signal-to-noise ratio, while still proving insufficient to meaningfully eliminate residual susceptibility artefacts. The same applies to the more advanced artefact reduction techniques envisaged for other metal implants – namely, view angle tilting (VAT), slice encoding for metal artefact correction (SEMAC), and multiacquisition variable-resonance image combination (MAVRIC) (Koch et al., 2010), which are similarly unsuitable for the use in the inherently sensitive brain tissue.

Susceptibility artefacts can be reduced by eliminating slice-selective and readout encoding processes, i.e. utilising full phase-encoding of the images, as is used in single point imaging techniques. One of the more promising single-point imaging techniques is single point ramped imaging with T_1_ enhancement (SPRITE) (Balcom et al., 1996), which exhibits substantial tolerance to magnetic susceptibility variations and eddy currents. At the expense of protracted imaging times despite the proposed use of ramped gradients, this sequence can offer impressive spatial fidelity by providing encoding immunity to B0 perturbations in the vicinity of metallic implants (Ramos-Cabrer et al., 2004). Yet, its clinical viability is considerably constrained by challenges as complicated volume selection and associated aliasing problems, hardware requirements and the need for excessive levels of acquisition acceleration with potentially deleterious effects on the final signal-to-noise ratios. Ergo, no viable application of the aforementioned artefact reduction technique in fMRI may be expectable in the near future.

**Fig. 4.**
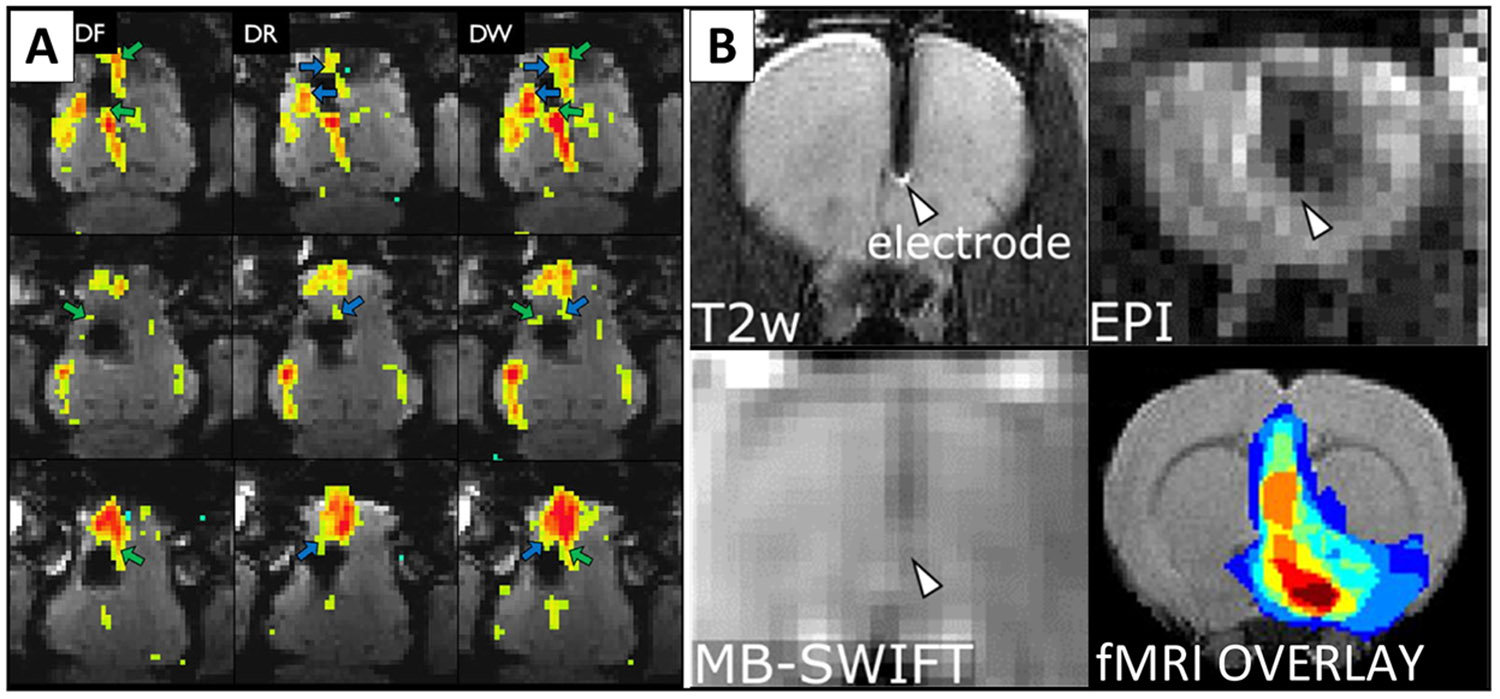
Viable mitigation strategies: A) Post-processing strategy to limit susceptibility artefacts around implanted DBS electrode using point spread function mapping (In et al. 2017). Blood oxygen level dependent contrasts of DBS stimulation effects as calculated from forward (DF) and reverse (DR) echo-planar imaging (EPI) sequence with distortion correction, combined into the weighted average (DW). DW exhibits substantially stronger effects than individual EPI scans, with green and blue arrows indicating only forward or reverse EPI scan findings. (Reprinted with permission, from reference In et al. 2017). **B) Susceptibility artefact around tungsten electrode implanted in rat brain** in T_2_-weighted (T2w) fast spin-echo sequence, echo-planar imaging (EPI) sequence, multi-band sweep imaging with Fourier transformation (MB-SWIFT) sequence, showing signal virtually devoid of magnetic susceptibility artefact in MB-SWIFT when compared to EPI. The lower right quadrant contains the fMRI group activation overlap map depicting high-quality MB-SWIFT fMRI signal in the vicinity of electrode, overlaid over a structural scan template of rat brain (Lehto et al., 2018). CC BY 4.0.

Another strategy to achieve spatially accurate MRI in the vicinity of implants lies in postprocessing applications following the acquisition of conventional MRI scans. Unfortunately, standard approaches to mitigate the distortion in the phase-encoding direction of EPI sequences are not readily applicable in situations involving DBS and other implants due to the challenge of generating spatially accurate field maps which generally assume only gradual spatial variation in frequency offsets of spin isochromats (Jezzard and Balaban, 1995). Although there has been some success (Skare and Andersson, 2005) with a postprocessing technique based on acquiring two images with opposing polarities of encoding gradients (Chang and Fitzpatrick, 1992), it is generally effective for SE and GE-EPI sequences primarily in the absence of metallic implants. When metal hardware is present, the algorithms based on distortion transformation mathematics become ill-conditioned (Koch et al., 2010), as excessive through-pixel frequency variations lead to the breakdown of the correction mechanism. Additionally, conservative SAR limitations in DBS fMRI present an additional obstacle. By contrast, a more recent development using point spread function mapping has shown promise for minimising metal-induced susceptibility artefacts directly in DBS fMRI (In et al., 2017) (see Fig. 4A). Although not entirely devoid of the peri-electrode artefacts, the use of a 3T clinical scanner with reasonable fMRI acquisition parameters and temporal requirements and further refinements into multi-band interleaved reverse-gradient fMRI (In et al., 2023) offer substantial potential for advancing data acquisition in regions subject to susceptibility-induced signal loss.

Considerable shortening of echo time in UTE sequences, or the use of zero echo time (ZTE) sequences, show significant promise, not only in general neuroimaging in DBS patients, but also in research applications. ZTE sequences are, in essence, pulse-acquire free induction decay (FID) pulse sequences which do not generate echo signals per se. While their most evident benefit in DBS imaging is the marked reduction in geometric distortions arising from variations in magnetic susceptibility (Yuan et al., 2020), the concomitant attenuation of acoustic noise – commonly related to gradient switching between excitations – is another notable advantage for patient comfort. The primary native contrast of ZTE sequences is proton density-weighted with a degree of T_1_ saturation, making them particularly suitable for structural imaging (Ljungberg et al., 2021). Nonetheless, T_2_ contrast can still be achieved by incorporating a T_2_ preparation module, typically consisting of a tip-down pulse shifting the magnetisation into the transverse plane (Deichmann et al., 1995). In practice, however, attaining functional BOLD contrast with such a ZTE sequence has proven challenging due to protracted acquisition times (Solana et al., 2016).

The recently introduced Looping Star ZTE sequence generates T_2_* contrast in the steady state, representing a practical surrogate for the GE-EPI sequences currently utilised in DBS fMRI protocols (Wiesinger et al., 2019). Its utility has been demonstrated across a variety of paradigms, including resting-state, motor and visual working memory tasks (Wiesinger et al., 2019, Dionisio-Parra et al., 2020), with further developments in the form of multi-echo capabilities to mitigate physiological noise (Damestani et al., 2021). Regrettably, in the current configuration, its SAR estimations substantially hinder its implementation in DBS fMRI.

An entirely distinct perspective on functional brain activity assessment has been recently demonstrated by ZTE-like sequences in the form of Sweep Imaging with Fourier transformation (SWIFT) (Idiyatullin et al., 2006) and Multi-Band SWIFT (MB-SWIFT) (Idiyatullin et al., 2006, Idiyatullin et al., 2015). In contrast to conventional T_2_*-weighted BOLD signal, SWIFT fMRI signals likely reflect increased blood flow during neuronal activity rather than changes in blood oxygenation (Lehto et al., 2017; Mangia et al., 2026). Studies have shown SWIFT-based fMRI to be a viable strategy not only in animal models (Lehto et al., 2017, Paasonen et al., 2022), but also in humans (Mangia et al., 2012). Most importantly though, MB-SWIFT has proven an exceptional strategy for alleviating susceptibility artefacts in DBS fMRI (Lehto et al., 2018) and in combined fMRI – electroencephalography (EEG) studies (Paasonen et al., 2020). In addition to improving image quality in the vicinity of electrodes relative to common EPI sequences (Fig. 4B), SWIFT substantially decreases current induction in DBS electrodes due to low gradient switching rate. Nonetheless, its thermogenic properties currently still exceed the recognised safety limits for human DBS MRI imaging, and the stringent requirements for RF transmit-receive switching present another significant barrier for its implementation outside high-level research facilities. Similar considerations extend to UTE-based sequences, recently safely employed in humans (Ponticorvo et al., 2024), although not in DBS settings so far. Recent preclinical advancements in UTE, ZTE, and MB-SWIFT in rats are promising in terms of adaptation these sequences for human fMRI paradigms (Lehto et al., 2017, Paasonen et al., 2022, Paasonen et al., 2020, Valjakka et al., 2025, Jain et al., 2026, Paasonen et al., 2022, Laakso et al., 2021).

## 5. Functional MRI in DBS: future prospects

RF-induced heating remains the principal risk associated with MRI acquisitions in patients with DBS and constitutes the major obstacle to the developments of further mitigation strategies, which, while relevant from clinical and research perspective, must remain secondary to the safety considerations. Although technically possible, the fundamental redesign of DBS hardware on the scale discussed in this review would require extensive research over many years to translate these currently preclinical concepts into clinically and commercially viable products. A seemingly straightforward approach to the heating issue – coiling the extension wire close to the burr hole – may, however, conflict with the efforts to reduce the extent of susceptibility artefacts from electrode wiring. Consequently, the most readily implementable solutions pertain to the innovations in MRI acquisition protocols.

Conventional fMRI sequences have yielded remarkable insights into DBS as a therapeutic modality generally restoring activation and connectivity patterns closer to those observed in healthy population (Loh et al., 2022, Jech and Mueller, 2022). Nonetheless, the notional holy grail would be the incorporation of this modality into routine clinical workflows already at the programming stage. The empirical search of optimal stimulation parameters as currently standard in the clinical practice usually necessitates multiple lengthy sessions. This practice not only strains healthcare resources, but might also pose mild to moderate discomfort to patients due to adverse effects associated with suboptimal stimulation, delays in achieving maximal therapeutic benefit and burdensome travel requirements given the general scarcity of advanced clinical centres offering this therapeutic option. Various semi-automated programming approaches have been proposed, utilising anatomical position of individual electrode contacts within the target region, its combination with other sensor metrics or clinical outcomes, as well as DBS effects on fMRI connectivity pattern (Cole and Miocinovic, 2025). Complex studies employing repeated fMRI acquisitions under different DBS settings are ambitious goals that could directly benefit patients (Boutet et al., 2021, Qiu et al., 2024). Nevertheless, several broader questions beyond the signal dropouts remain open. These include the reproducibility of functional responses, the identification of appropriate metrics and machine learning algorithms, and finally whether the common ‘therapeutic network’ exists across individuals. One of the major hurdles lies in the loss of signal in regions highly relevant for the programming efforts – not only the implantation site itself, but also extensive portions of sensorimotor cortex (see Fig. 1).

The strategies highlighted in this review have the potential to alleviate this constraint. Advanced post-processing algorithms based on point spread function mapping have already been validated in DBS fMRI (In et al., 2017). Although not able to remove susceptibility artefacts around DBS hardware in their entirety, they can substantially extend the regions where high-quality signal can be obtained and are readily adaptable to existing MRI protocols (see Fig. 4A). Approaches that are nearly impervious to spatial distortions around DBS electrodes such as SWIFT (Idiyatullin et al., 2006) hold even more promise (see Fig. 4B). Substantial further developments, however, will be required to render the sequence clinically viable and safe for DBS patients. Nonetheless, the ability to acquire information from the very core of the signal “black hole” around the DBS electrode is likely to prove invaluable in elucidating more intricate, local DBS effects. Moreover, a broad range of current fMRI research would benefit from the aforementioned advances for three reasons: 1. Even in large-scale network analyses, regions in the vicinity of the electrode may constitute important components of distributed brain networks 2. Electrode artefact mitigation strategies are also applicable to other forms of susceptibility artefacts, for example susceptibility artefacts around air-tissue interface, 3. Envisioned sequences such as MB-SWIFT are 3D sequences that are less affected by motion artefacts, and the RF pulses utilized are less prone to B1 inhomogeneities (Idiyatullin et al., 2015). Last, but not least, SWIFT’s established excellent compatibility with simultaneous scalp EEG, due to slower gradient switching leading to lower induced current EEG noise artefacts (Paasonen et al., 2020). Dual EEG-SWIFT fMRI recordings have the potential to provide a promising extension of current DBS-fMRI literature, supporting both resting-state and task-based fMRI designs with high temporal resolution EEG information. Multimodal acquisitions may ultimately help characterize and reduce sources of variability and allow for more informed inferences on underlying biology (Zuo et al., 2014). By adopting meticulous study design with emphasis on retest reliability and clearly formed hypotheses, augmenting cohort sizes through multi-centric data, managing susceptibility artefacts through innovative MRI sequence designs, and integrating complementary modalities such as combined fMRI-EEG and multi-faceted clinical response data, it will be possible to deepen our understanding of DBS mechanisms and refine DBS programming practices.

## 6. Conclusion

fMRI in DBS patients holds the promise not only of revealing the broader underpinnings of neurostimulation effects at the general level of DBS targets and neuropathologies, but also of capturing individual, patient-specific responses. This capacity could, in turn, lead to more efficient programming and avert the unwanted stimulation of regions potentially associated with adverse effects. This review outlines several mitigation strategies of known fMRI DBS challenges related to safety and signal quality, ranging from innovative DBS hardware solutions to advanced MRI sequences designed to address problems inherent to the presence of DBS hardware. Our purpose is to encourage continued research towards refining and disseminating MRI and fMRI protocols to wider research communities and ultimately into the clinical practice, with the overarching objective of improving the quality of life of DBS patients.

## Figures and Tables

**Table 1:** Manufacturer guidelines on RF field strength (Abbott Ap 2025, Boston Scientific M 2026, Medtronic, 2022).

Manufacturer		B_1+RMS_ requirement		SAR requirement
		1.5T	3T	
Medtronic	Directed lead	≤2.0 μT; ≤1.7 μT if the neurostimulator is implanted in the abdomen	≤2.0 μT for Percept neurostimulator	Whole body ≤0.1 W/kg
Nondirected lead	≤2.0 μT	≤2.5 μT for Percept neurostimulator		
Abbott		Head ≤1.8 μT, whole body ≤1.1 μT (Torso)	Not compatible	Head ≤0.3 W/kg, whole body ≤0.1 W/kg
Boston Scientific		≤2.0 μT	Not compatible	Whole body ≤0.2 W/kg or ≤0.1 W/kg depending on neurostimulator

## Abbreviations

B_1_: radiofrequency magnetic field
B_1+rms_: root-mean-square of the radiofrequency magnetic field
BOLD: blood oxygen level dependent
DBS: deep brain stimulation
EPI: echo planar imaging
fMRI: functional magnetic resonance imaging
GRE-EPI: gradient recalled echo echo-planar imaging
MAVRIC: multiacquisition variable-resonance image combination
MB-SWIFT: multi-band sweep imaging with Fourier transformation
MPRAGE: T_1_-weighted magnetisation-prepared rapid gradient echoes
MRI: magnetic resonance imaging
RF: radiofrequency
SAR: specific absorption rate
SE: spin-echo
SEMAC: slice encoding for metal artefact correction
SPRITE: single point ramped imaging with T_1_ enhancement
STIR: short-tau inversion recovery
SWIFT: sweep imaging with Fourier transformation
T_2_w: T_2_-weighted
TE: echo time
UTE: ultra-short echo time
VAT: view angle tilting
ZTE: Zero Echo Time
